# Loss of Both CDKN2A and CDKN2B Allows for Centrosome Overduplication in Melanoma

**DOI:** 10.1016/j.jid.2020.01.024

**Published:** 2020-09

**Authors:** Shyamal Patel, Christopher J. Wilkinson, Elena V. Sviderskaya

**Affiliations:** 1 Cell Biology Research Centre, Molecular and Clinical Sciences Research Institute, St. George's, University of London, Cranmer Terrace, London, United Kingdom; 2 Centre for Biomedical Sciences, Department of Biological Sciences, School of Life Sciences and the Environment, Royal Holloway University of London, Egham, United Kingdom

**Keywords:** RGP, radial growth phase, siRNA, small interfering RNA, VGP, vertical growth phase

## Abstract

Centrosomes duplicate only once in coordination with the DNA replication cycle and have an important role in segregating genetic material. In contrast, most cancer cells have centrosome aberrations, including supernumerary centrosomes, and this correlates with aneuploidy and genetic instability. The tumor suppressors p16 (CDKN2A) and p15 (CDKN2B) (encoded by the familial melanoma *CDKN2* locus) inhibit CDK4/6 activity and have important roles in cellular senescence. p16 is also associated with suppressing centrosomal aberrations in breast cancer; however, the role of p15 in centrosome amplification is unknown. Here, we investigated the relationship between p15 and p16 expression, centrosome number abnormalities, and melanoma progression in cell lines derived from various stages of melanoma progression. We found that normal human melanocyte lines did not exhibit centrosome number abnormalities, whereas those from later stages of melanoma did. Additionally, under conditions of S-phase block, p15 and p16 status determined whether centrosome overduplication would occur. Indeed, removal of p15 from p16-negative cell lines derived from various stages of melanoma progression changed cells that previously would not overduplicate their centrosomes into cells that did. Although this study used cell lines in vitro, it suggests that, during clinical melanoma progression, sequential loss of p15 and p16 provides conditions for centrosome duplication to become deregulated with consequences for genome instability.

## Introduction

Extra centrosomes are frequently observed in solid tumors including melanomas, and this centrosome amplification correlates with aneuploidy and genetic instability (Brinkley, 2001, Charters et al., 2011, Lingle et al., 2002, Pihan et al., 1998). A mechanistic link between these organelles and tumorigenesis was first proposed by Boveri over a century ago (Boveri, 2008, Boveri, 1914). The abnormal spindle structures he predicted have been observed in clinical samples. They are eventually resolved into bipolar spindles, but the delay leads to lagging chromosomes that are easily damaged (Ganem et al., 2009). The centrosome is normally duplicated in S-phase and separated into two centrosomes in G2, with each centrosome contributing to one of the two spindle poles in M phase. A normal cell therefore contains a maximum of two centrosomes; any more are supernumerary.

The root cause of extra centrosomes in tumor cells has been the subject of debate for some time (Nigg, 2002). p53 deficiency has been shown to lead to centrosome amplification through excessive duplication of the existing component centrioles (Fukasawa et al., 1996, Prosser et al., 2009). It has also been proposed that in many cases cytokinesis failure, which is tolerated in p53-deficient cells, results in cells with tetraploid DNA and an extra centrosome from the previous cell cycle (Borel et al., 2002, Meraldi et al., 2002). However, such accumulated centrosomes do not persist in cell culture (Krzywicka-Racka and Sluder, 2011). Centrosome amplification—the presence of supernumerary centrosomes—has therefore been proposed to occur by two main routes, overduplication, in which the normal process of centriole duplication is repeated before cell division, giving four or more centrioles (two or more centrosomes), and accumulation, in which cells abort cell division sometime after S-phase so the cell arrests with double the chromosome as well as centrosome/centriole complement (Nigg, 2002). In melanoma, p53 mutations are significantly less common compared with the frequency observed in most other cancers so, in either case, the abnormal numbers of centrosomes in melanoma cannot be attributed to p53 loss (Fecher et al., 2007).

One example of genuine, clinical centrosome overduplication is in cervical carcinoma, of which 90% of cases are caused by human papillomavirus (zur Hausen, 1996). Oncogenic human papillomavirus encodes two proteins, E6 and E7, that effectively remove p53 and the retinoblastoma protein family of proteins, respectively (Duensing and Münger, 2003). Retinoblastoma protein inactivation has a direct effect on centrosome duplication through activation of the E2F transcription factor family and initiation of expression of S-phase proteins, including CDK2 and associated cyclins (Meraldi et al., 1999). CDK2-cyclin A/E is a powerful driver of centrosome duplication (Hinchcliffe et al., 1999, Meraldi et al., 1999). By selective molecular marking of old versus new centrioles, Duensing et al. were able to show that E7 expression causes centrosome overduplication (as opposed to centrosome accumulation) (Duensing et al., 2008, Duensing and Münger, 2003).

The *CDKN2* locus, located on chromosome 9p21, encodes three distinct tumor suppressor proteins: p16 (INK4A) encoded by *CDKN2A*, p15 (INK4B) encoded by *CDKN2B*, and p14^ARF^ (p19^ARF^ in mice) encoded by an alternative reading frame of *CDKN2A* (Hannon, 1994, Quelle et al., 1995). p16 and p14^ARF^ have mirror image roles to human papillomavirus E6 and E7. p16 binds to and inactivates CDK4/6, preventing retinoblastoma protein inactivation and cell cycle initiation (Serrano et al., 1993). p14^ARF^ binds to and inactivates MDM2, thus saving p53 from proteolytic degradation (Pomerantz et al., 1998, Sherr and Weber, 2000). Together, the two proteins act as brakes on the cell cycle (Bennett, 2003, Sviderskaya et al., 2003). Loss of p16 leads to an apparent increase in centrosome number, but this has been ascribed to splitting of the centrosome into separate centrioles (McDermott et al., 2006). Similar to p16, p15 is involved in inhibiting CDK4/6 by binding to a noncatalytic site of these kinases (Krimpenfort et al., 2007, Pavletich, 1999). Mice deficient for all three genes encoded by the *CDKN2* locus develop a wider spectrum of tumors than those lacking only p16 and p19^ARF^.

Loss of either the *CDKN2A* locus or the entire *CDKN2* cluster is frequently observed in human cancers (Orlow et al., 1999). It is particularly prevalent in melanomas (Bennett, 2003). Sequential loss of alleles encoding p16 has been proposed to contribute to the progression from normal melanocytes to malignant melanoma via benign nevi, dysplastic nevi, radial growth phase (RGP), and vertical growth phase (VGP) stages (Bennett, 2008, Ha et al., 2008).

The close mechanistic link between these genes, known to be mutated in cancer, and the centrosome duplication pathway would suggest that their loss may be responsible for the extra centrosomes seen in melanoma. Decreased p16/15 levels would result in increased CDK4/cyclin D activity, reduced inhibition of E2F by retinoblastoma protein, and increased CDK2 activity, driving centrosome overduplication. It would follow that centrosome numbers should also increase with melanoma progression as p16 and/or p15 expression is progressively lost.

The resources of the Wellcome Trust Functional Genomics Cell Bank at St George’s, University of London, offered a good opportunity to test these two hypotheses in melanoma-derived cell lines. Although these cell lines are not primary cells from melanomas, they will be very close in genotype to such cells and serve as a good model for clinical melanoma.

## Results

### Centrosome numbers at different stages of tumorigenesis

To find out if cells from later stages of melanoma would display increased centrosome numbers, cells were stained with an anti–γ-tubulin antibody that binds to the material around each centriole, each centrosome therefore showing as two punctae of staining.

**Figure 1:**
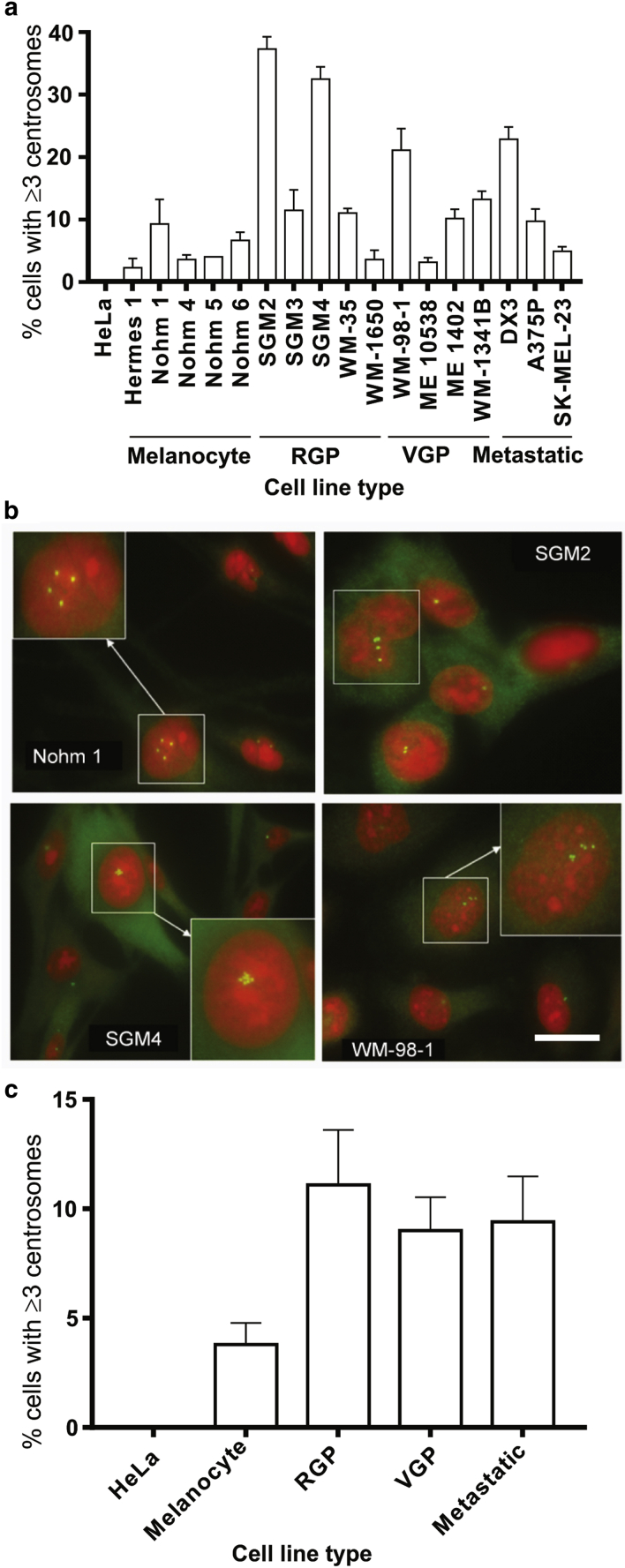
**Centrosome abnormalities in cell lines from different stages of melanoma progression.** (**a**) Bar graph showing the percentage of cells with three or more centrosomes in melanocyte/RGP/VGP and metastatic melanoma lines. At least 100 cells were analyzed per experiment. (**b**) Immunostaining of centrosomes (γ-tubulin, green) and nuclei (propidium iodide, red) in melanocyte cell line Nohm 1, RGP cell lines SGM2 and SGM4, and VGP cell line WM-98-1. Insets show centrosomes magnified. Bar = 10 μm. (**c**) Bar graph representing combined data from (a) showing the proportion of centrosome number abnormality (three or more centrosomes). Mean percentage of cells with two or fewer and three or more centrosomes across five melanocyte lines, five RGP lines, four VGP lines, and three metastatic melanoma lines. Statistical significance is marked as ∗*P* < 0.05, ∗∗*P* < 0.01, and ∗∗∗*P* < 0.001. The level of statistical significance shown is that relative to the % of cells in melanocyte lines for each category (two or fewer and three or more centrosomes). RGP, radial growth phase; VGP, vertical growth phase.

Figure 1a shows the percentage of cells that expressed an abnormal (>3) number of centrosomes across melanocyte, RGP, VGP, and metastatic melanoma lines. Centrosome amplification was observed in several cell lines, notably RGP lines SGM2 and SGM4 (Figure 1b), nearly a quarter of whose cells had three or more centrosomes. No melanocyte lines displayed substantial centrosome amplification. As a set, melanoma cell lines had higher levels of centrosome amplification than melanocytes (Figure 1c). However, all stages of melanoma progression included cell lines where very few cells had supernumerary centrosomes.

### Ploidy of cells displaying centrosome amplification

To distinguish between the two routes to centrosome amplification—overduplication and accumulation—this study compared the DNA content of cells that displayed centrosome amplification with those from the same cell line that did not, by quantification of propidium iodide staining, the analysis used by Meraldi et al. (2002).

**Figure 2:**
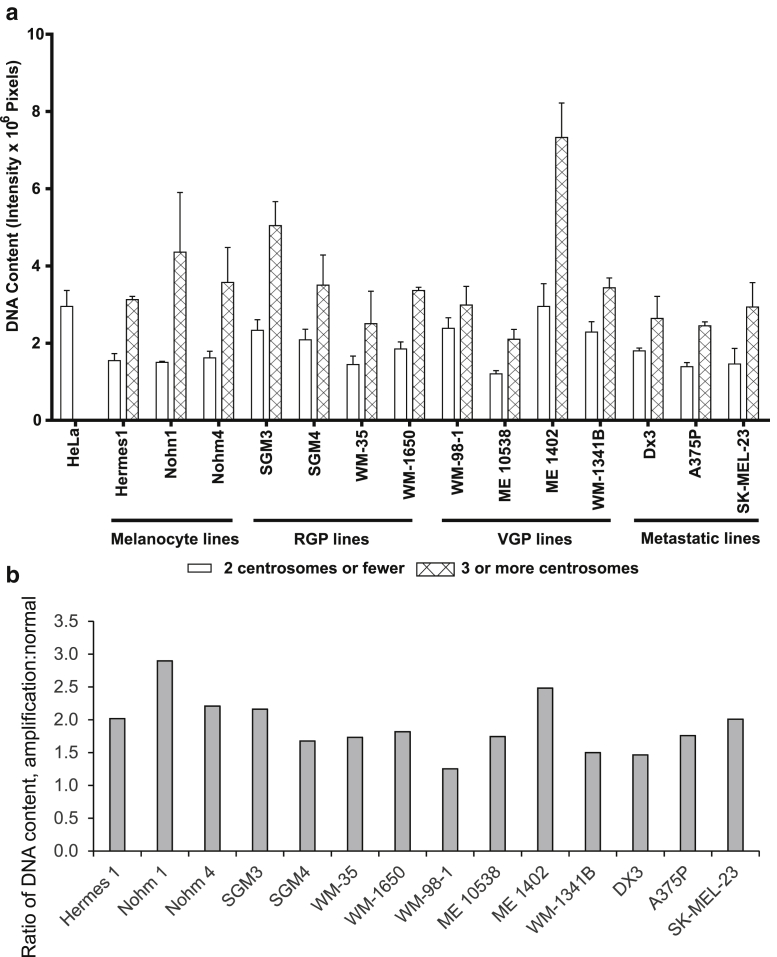
**Relationship between centrosome number and DNA content.** (**a**) Comparison of DNA content between cells with two or fewer and three or more centrosomes in cell lines from different stages of melanoma progression. Propidium iodide was used to stain nuclei. The nuclear area of each cell (at least 100 cells for each cell line) was analyzed to determine the differences in propidium iodide fluorescence intensity between the two groups of cells. (**b**) Ratio of DNA content in cells with centrosome amplification to normal cells, using DNA content from (a). RGP, radial growth phase; VGP, vertical growth phase.

Of the 17 cell lines examined, all showed significantly higher DNA content in cells with three or more centrosomes than in those with two or one (Figure 2). If we calculate the ratio of DNA content of cells with centrosome amplification to normal cells, we see that all but one line have ratios in the range 1.5×–2.5×. Six lines have a ratio of 2× or above. In these cases, we can confidently assign the increase in centrosome number to accumulation. In the other lines, the increase in DNA content could be due to a dividing subpopulation that has acquired and then reduced a tetraploid DNA complement while retaining some excess centrosomes.

### p15 and p16 expression in cell lines from different stages of melanoma progression

**Figure 3:**
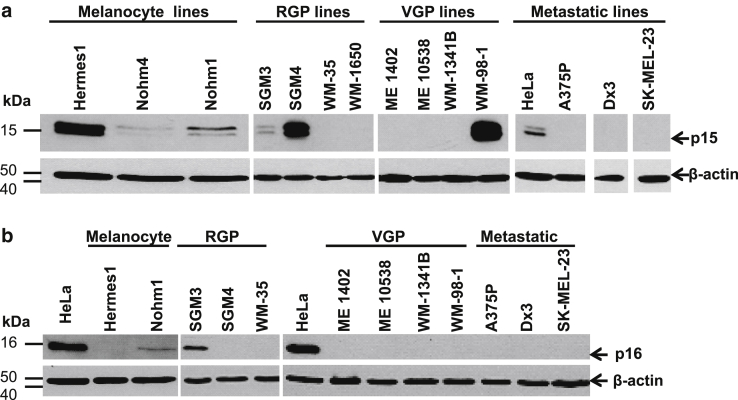
**p15 and p16 expression in cell lines.** (**a**) Western blotting analysis of p15 and (**b**) p16 expression in melanocyte, RGP, VGP, and metastatic melanoma lysates, respectively. A total of 30–40 μg protein per well was resolved from each sample. HeLa cell line represented a positive and negative control, respectively. β-actin was probed as a loading control on all blots. RGP, radial growth phase; VGP, vertical growth phase.

In case there was a relationship between centrosome amplification and p15 and/or p16 status rather than melanoma stage, we determined by western blotting the p15 and p16 levels in all cell lines used (Figure 3). The normal (Nohm) melanocytes, which will eventually senesce, were all positive for both p15 and p16 but expression was relatively low, expected for passage number 15–25. Either p16 alone or both p15 and p16 together were lost from premetastatic melanoma lines. Even at the RGP stage, some lines had lost expression of both proteins. All metastatic lines were double negative for p15 and p16 (p15^−^p16^−^). Progressive loss of p15 and p16 is therefore observed as melanoma develops, but tumor stage is not an absolute predictor of p15 and p16 status, and neither is p15 and p16 status a predictor of whether centrosome amplification is observed.

### Susceptibility of cells to centrosome overduplication in relation to their p15/p16 status

Hydroxyurea treatment of cells, which imposes an S-phase block, causes centrosome overduplication in a number of cell lines and is the basis of the centrosome duplication assay that has been used to examine how centrosome duplication is controlled (Balczon et al., 1995, Habedanck et al., 2005). We sought to test if p15/p16 status had an effect on whether melanoma cell lines would overduplicate centrosomes in this assay. We confirmed that hydroxyurea treatment caused an S-phase block in these two cell lines by FACS analysis (data not shown).

**Figure 4:**
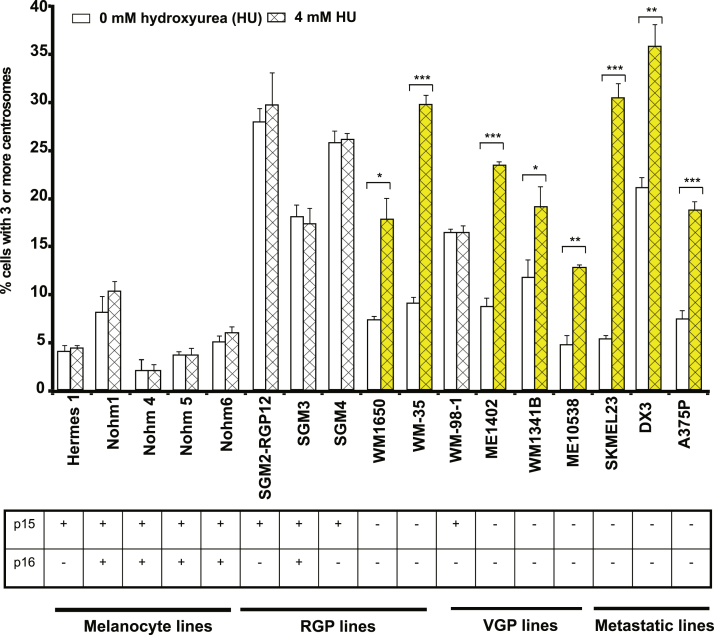
**Results of the centrosome duplication assay, using melanoma cell lines.** Effect of HU-induced S-phase block (4 mM for 48 hours) on centrosome duplication for all cell lines. Statistical significance is marked as ∗∗∗*P* < 0.001, ∗∗*P* < 0.01, and ∗*P* < 0.1. The table indicates the p15 and p16 expression status (**+**: cell line expressing p15 or p16; **–**: cell line null for p15 or p16). HU, hydroxyurea; RGP, radial growth phase; VGP, vertical growth phase.

All normal melanocyte lines—Nohm1, Nohm 4, Nohm 5, and Nohm 6, all p15- and p16-positive—showed no significant change in the proportion of cells with centrosome number abnormalities (Figure 4). All four p15^+^p16^–^ cell lines also showed no change in frequency of cells, with at least three centrosomes in the centrosome duplication assay.

All eight p15 and p16 double negative lines overduplicated centrosomes (yellow in Figure 4). Untreated cells displayed a low level of centrosome amplification, whereas hydroxyurea-treated cells displayed a significant increase in centrosome numbers. This strongly suggests that both p15 and p16 need to be absent for the centrosome duplication and DNA replication cycles to be uncoupled and centrosome overduplication to occur.

### Effect of altering p15 and p16 expression levels on centrosome overduplication

If loss of p15 is important for centriole overduplication, provided p16 is also absent, then depletion of p15 from p15^+^p16^–^ cells should result in overduplication, whereas expression of p15 in p15^–^p16^–^ cells should inhibit it.

**Figure 5:**
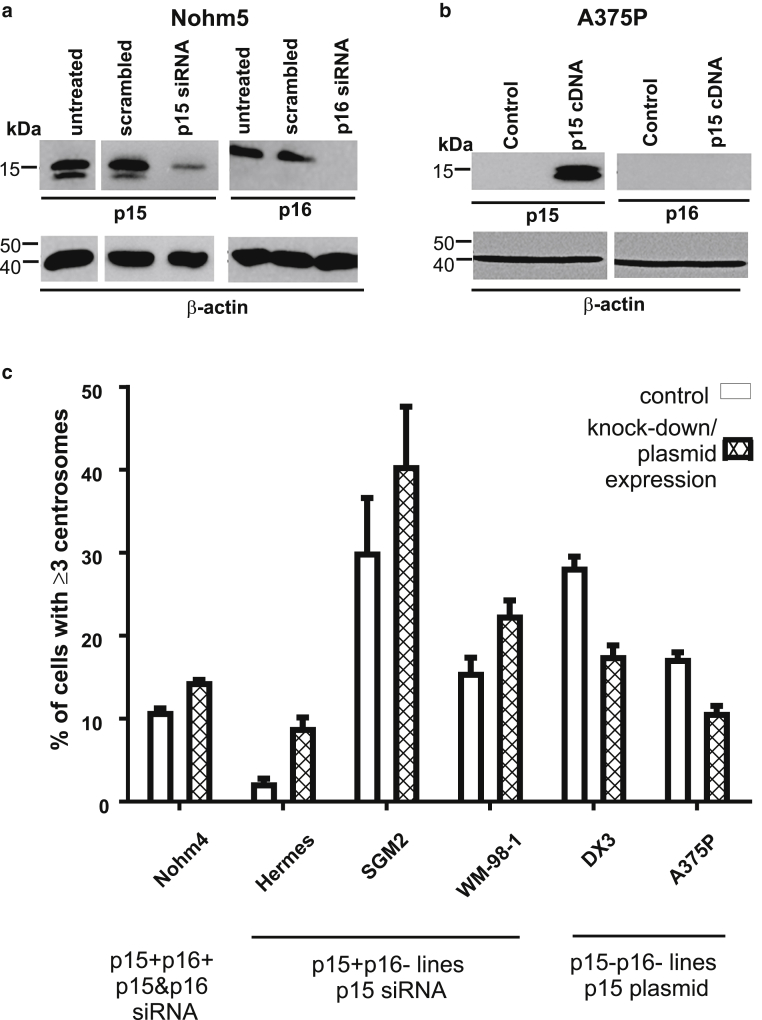
**Effect of altering p15/p16 levels on the outcome of the centrosome duplication assay.** Depletion or restoration of expression of p15/p16 by transient transfection using *CDKN2A/B* siRNA or expression plasmid and its effect on centrosome duplication. (**a**) Western blotting analysis of p15 (left) and p16 (right) expression in A375P melanoma cells after transfection with p15 expression construct pBabe-puro-hp15. (**b**) Western blotting analysis of p15 and p16 expression in Nohm5 cells post-transfection with respective siRNAs (72 hours). (**c**) Centrosome (over)duplication in melanoma lines depleted of or complemented with p15 (and p16). Nohm4 cells were depleted of both p15 and p16. Hermes1, SGM2, and WM-98-1 cells were depleted of p15. A375P and DX3 cells were complemented with p15 by transfection with a plasmid encoding p15. All differences statistically significant at *P* < 0.05 or less. siRNA, small interfering RNA.

We depleted p15 and p16 by small interfering RNA (siRNA), judging efficacy by western blotting of cell extracts from Nohm5 cells transfected with respective siRNAs (Figure 5a). We complemented loss of p15 by transfection of cells with an expression construct for p15. This was judged effective by western blotting of extracts from A375P cells treated in this way.

In the case of p15^+^p16^–^ cells now depleted of p15, centrosome numbers increased significantly during the centrosome duplication assay (Figure 5c), although the level of overduplication in Hermes 1 was modest. In the case of p15^–^p16^–^ cells now complemented in trans with p15, centrosome overduplication was now significantly inhibited (Figure 5c). This was true both in the case of a line carrying the BRAF oncogene, A375P, and a line carrying the NRAS Q61K oncogene, DX3. Nohm4 cells, depleted of both p15 and p16 by siRNA, showed a very modest increase in centrosome overduplication. In all cases, results were consistent with the proposal that, for centrosome overduplication to occur, both p15 and p16 need to be absent.

### Mechanism behind supernumerary centrosomes

**Figure 6:**
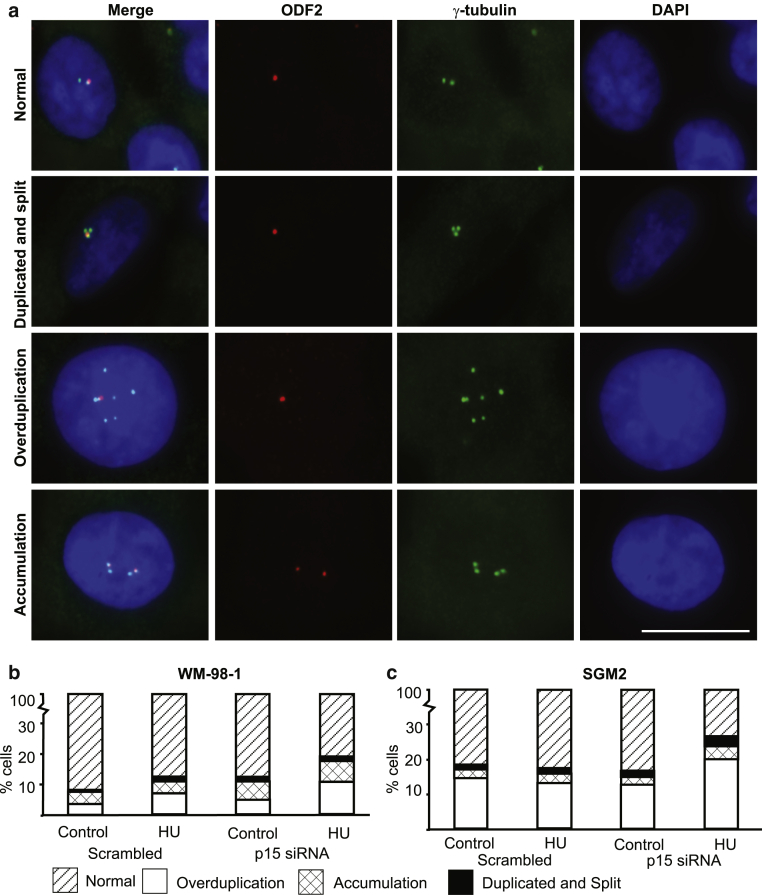
**Type of centrosome amplification observed in p15- p16-depleted cells.** (**a**) Centrosome amplification in p15- and p16-depleted cells. ODF2 (red, mother centriole) and γ-tubulin (green, both centrioles) staining of centrosomes in normal cells and abnormal cells (duplicated and split, overduplicated and accumulated centrosomes). The nuclei are stained with DAPI (blue). (**b, c**) Nature of centrosome amplification in p15- and p16-depleted cells. Bar graphs of normal versus duplicated and split versus overduplicated versus accumulated centrosomes in WM-98-1 (b) and SGM2 (c) cell lines post–siRNA transfection followed by the centrosome duplication assay. HU, hydroxyurea; siRNA, small interfering RNA.

Centrosome amplification could result from one of three processes: accumulation (after failed division), overduplication, and premature splitting of a duplicated centrosome. Following the example of Duensing et al. (2008), we stained mother centriole appendages to distinguish between these three outcomes, using an antibody to ODF2 (Lange and Gull, 1995) (Figure 6a, first row).

According to the number and staining of fluorescent puncta, cells with supernumerary centrosomes were classified as follows: split after duplication, one punctum of ODF2 with three or four separate puncta of γ-tubulin (Figure 6a, second row); overduplication, one punctum of ODF2 with five or more puncta of γ-tubulin (Figure 6a, third row); accumulation, two puncta of ODF2 with four or more puncta of γ-tubulin (Figure 6a, fourth row).

We observed only a small proportion of cells with duplicated and split centrosomes (Figure 6b and c). Treatment with siRNA to p15 or p16 alone or both had no effect on the level of duplicated and split centrosomes compared with that seen in control (scrambled) siRNA-treated cells. Overduplication accounted for all the extra centrosomes observed (Figure 6b and c).

## Discussion

Expression of *CDKN2* is frequently lost in human melanoma. Moreover, in approximately 20% of all melanomas, p16 is expressed but remains inactive because of mutations (Bennett, 2016). We examined cell lines derived from different stages of melanoma progression. Melanocyte lines were positive for both p15 and p16 but at low levels. However, RGP and VGP melanomas varied in p15 and p16 status. RGP lines were a mixture of p15-positive and p16-negative (p15^+^p16^–^), p15 and p16 double positive (p15^+^p16^+^), and p15 and p16 double negative lines (p15^–^p16^–^). Almost all VGP and all metastatic lines had lost expression of both proteins.

Supernumerary centrosomes are frequently observed in human cancers. It is possible that they arise either owing to cytokinesis failure and accumulation of extra centrosomes or owing to deregulated centrosome duplication. Although we observed a step increase in the proportion of cells with extra centrosomes from normal melanocytes to the various melanoma lines, we found that the expression status of p15 and p16 per se cannot predict the degree of centrosome supernumerary aberrations in cell lines from each stage of melanoma progression. Indeed, it appears that the supernumerary centrosomes observed in many cultured lines were due to mitotic failure leading to centrosome accumulation alongside tetraploidy. Indeed, RGP cells seem to go through a crisis before progressing the VGP stage, with anaphase bridges and mitotic failures, which fits with these results (Bennett, 2016, Soo et al., 2011). This might explain the step-change in centrosome numbers between normal melanocytes and melanoma cells of all stages, the first of which is RGP.

p15/p16 status did affect whether these cells overduplicated centrioles under the centrosome duplication assay that has been extensively used to determine the contribution of centrosome proteins and cell cycle regulators. For centrosome overduplication to occur, both p15 and p16 needed to be absent. The presence of either or both prevents overduplication. This effect is independent of the oncogene present, as lines carrying either BRAF or NRAS oncogenes behaved in the same way. In assay conditions, genuine overduplication is observed as opposed to the accumulation seen in continuous culture. It is notable in this regard that it has been reported that the extra centrosomes observed in melanoma biopsies are due to overduplication rather than accumulation (Denu et al., 2018) and so are closer to our assay results. This suggests that p15 and p16 may have a similar effect in melanoma.

The response of many widely used model cell lines to this centrosome overduplication assay varies. Some, such as U2OS, overduplicate centrioles, whereas others, such as HeLa, do not. Although hydroxyurea treatment should induce an S-phase block by inhibiting DNA synthesis, Khodjakov has proposed that cells that overduplicate centrioles under these conditions slip into a G2-like state rather than an S-phase arrest (Loncarek et al., 2010). In such cells, high PLK1 activity in G2 allows procentrioles to mature, disengage from parent centrioles, and subsequently reduplicate. Fry et al. showed that anaphase promoting complex/cyclosome and CDK2 activities cycle under these conditions (under G2 arrest, PLK1 degrades the anaphase promoting complex/cyclosome inhibitor, thus activating anaphase promoting complex/cyclosome), leading to rounds of centriole disengagement and duplication (Prosser et al., 2012). The question still remains why some cells such as U2OS slip into this quasi-G2 state, whereas others such as HeLa stay blocked in S-phase.

The status of p15/p16 expression could be relevant to this question. Previously, it was shown that p16 overexpression in U2OS cells suppresses overduplication under hydroxyurea block (Meraldi et al., 1999). This overexpression is also complementing the genetic lesion in these cells, which are p16-negative (data not shown). In contrast, HeLa cells are often used as a positive control for *CDKN2* expression as this line is both p15- and p16-positive (Figure 3). Because HeLa cells will overduplicate centrioles when regulators such as PLK4 are overexpressed in these cells or blocked in G2 (Loncarek et al., 2010, Meraldi et al., 1999), this implies the limiting factor is upstream in the centriole duplication pathways or earlier in the cell cycle. p15 and p16 activity could be that factor. Loss of p15 and/or p16 would result in increased activity of CDK4/6-cyclinD, whose protein levels are maintained throughout the cycle, and could provide the drive to push cells into a quasi-G2 state in which the centrosome duplication cycle continues irrespective of the blocked DNA cycle. There are reports showing that CDK4/6-cyclinD plays a role in progression through G2 and contributes, with CDK2, to centrosome duplication (Adon et al., 2010, Gabrielli et al., 1999).

In tumor-derived cells, one would not expect p15/p16 status to be the only determinant of centrosome overduplication but a brake that needs to be off for overduplication to occur. Accordingly, centrosome overduplication must depend on other oncogenic changes but require p15 and p16 loss. Interplay between the CDKN2 proteins and other key drivers of melanoma progression such as BRAF and NRAS mutations (Davies et al., 2002) must be required for centrosome overduplication. Indeed, many of the cell lines in this study are known to have the following oncogenic mutations: BRAF V600E in the SGM2-4 and A375P lines or NRAS Q61K in the SGM5 and DX3 lines (Soo et al., 2011). Furthermore, we sequenced the relevant genomic fragments covering these mutations in the WM-98-1 line and found it to possess the BRAF V600E mutation (Supplementary Table S1). Therefore, the SGM2, WM-98-1, and A375P lines all possess the BRAF V600E mutation and both overduplicate centrioles when p15 and p16 are absent. However, this effect is not limited to the BRAF oncogene, as we obtain the same results with DX3 cells that carry the NRAS oncogene. Future work to test the hypothesis that the combination of BRAF or NRAS oncogenes and p15/p16 loss is sufficient for centrosome overduplication to occur will necessitate the creation of new stable cell lines, derived from normal melanocytes, that harbor this combination of mutations but lack the other mutations that may be present in melanoma lines.

In melanoma, mutations, deletions, or silencing of the *CDKN2A* locus often results in loss of both p16 and p14^ARF^. Loss of p14^ARF^ affects p53 stability via increased MDM2 activity, although p14^ARF^ is also known to have p53-independent mechanisms of action (Ha et al., 2007). p53 mutations in melanoma are themselves rare (Bennett, 2016, Gembarska et al., 2012). However, MDM4, another inhibitor of TP53, has been found to be upregulated in 69% of human melanomas (Gembarska et al., 2012) and in all the melanoma cell lines examined in that same study*.* Therefore, cells in which the entire *CDKN2* cluster is lost face the perfect storm for centrosome amplification. With centriole overduplication uninhibited and a reduced ability to respond to errors in centrosome duplication, supernumerary centrosomes can be generated and tolerated. Although our study has been conducted on cultured cell lines derived from melanomas, it suggests that, in melanoma, the very mutations that allow cells to escape normal growth controls, including cellular senescence, also allow centrosome duplication to become deregulated. It will be interesting to see if clinical samples of early melanomas also display correlation between loss of p15 and p16 activity and increased centrosome numbers. Such validation of this theory in clinical melanoma samples will need to be the subject of a future, more extensive study.

## Materials and Methods

### Cell culture

Human melanoma cell lines were grown in RPMI 1640 medium (Invitrogen, Carlsbad, CA) supplemented with fetal calf serum (10%, Invitrogen), L-glutamine (2 mM, Invitrogen), penicillin/streptomycin (100 U/ml and 100 μg/ml, respectively, Sigma, St. Louis, MO), and extra phenol red (7.5 μg/ml, Sigma) at 37 °C with 10% CO_2._ Human normal and immortal melanocyte lines were grown as described with the addition of 200 nM 12-O-tetradecanoyl phorbol 13-acetate (Sigma), 200 pM cholera toxin (Sigma), 10 ng/ml human stem cell factor (Invitrogen), and 10 nM endothelin 1 (Bachem, Bubendorf, Switzerland). To assay for centrosome duplication, fresh medium was supplemented with 4 mM hydroxyurea (Sigma) and cells were incubated for 48 hours before fixing.

### Immunostaining

Cells were fixed at 75% confluence in –20 °C methanol (Thermo Fisher Scientific, Waltham, MA) for 5 minutes then blocked with 1% BSA (Sigma) prepared in complete PBS for 20 minutes. Cells were probed with antibodies for 1 hour at room temperature. Primary antibodies used were against γ-tubulin (1:1,000, Sigma) and ODF2 (1:500, Abcam, Cambridge, United Kingdom). Secondary antibodies were Alexa Fluor 488 goat anti-mouse IgG and/or Alexa Fluor 594 goat anti-rabbit IgG (1:200, Invitrogen). The nuclei were stained with propidium iodide (1 μg/ml, Sigma) or DAPI (1 μg/ml, Invitrogen). Cells were mounted in Citifluor (Agar Scientific, Stansted, United Kingdom). Coverslips were washed with complete PBS between steps. Images were taken using an Axioplan 2 epifluorescence microscope and AxioVision 3 software (Carl Zeiss, Oberkochen, Germany). Images of cells stained with propidium iodide were further analyzed using the AxioVision 3 software to quantify DNA content for each nucleus.

### Western blotting

Cells were lysed using lysis buffer containing 50 mM Tris (pH 8), 150 mM NaCl, 2 mM EDTA, 1% Triton-X100, 1% SDS, and 1× protease inhibitor (Complete Mini Cocktail Tablets, Roche, Basel, Switzerland). The lysates were homogenized then centrifuged for 15 minutes at 6,613*g*. Protein concentration was determined using the Pierce BCA Protein assay kit (Thermo Fisher Scientific). A total of 30–40 μg of protein from each sample were resolved by a 15% polyacrylamide gel and wet transferred to a polyvinylidene fluoride membrane (Millipore). The membranes were blocked in PBS containing 5% dried milk powder and 0.1% Tween-20 for 1 hour at room temperature and then incubated in anti-p15 (1:1,000, Santa Cruz Biotechnology, Dallas, TX) or anti-p16 (1:1,000, Cell Signaling Technology, Danvers, MA) primary antibody overnight at 4 °C. Following incubation in horseradish peroxidase–conjugated anti-mouse (for p15) or anti-rabbit (for p16) secondary antibody (1:2,000, Cell Signaling Technology) for 1 hour at room temperature, proteins were detected using enhance chemiluminescence (GE Healthcare, Chicago, IL). A total of two bands for p15 were revealed as previously described by Fuxe et al. (2000). To reprobe the blots with a loading control antibody, the membranes were stripped for 10 minutes at room temperature in stripping solution (Millipore, Burlington, MA) and blocked for 1 hour before reprobing for β-actin (1:1,000, Cell Signaling Technology).

### Transfection of cultured cells

Nohm 4, WM-98-1, and SGM2 cells were transfected using DharmaFECT transfection reagent 2 (0.1 μl/100 μl well, Abgene, Portsmouth, NH) with ON-TARGET plus SMART pools of siRNAs directed against human *CDKN2A* and/or *CDKN2B* (25 nM) as per the manufacturer’s guidance. A375P cells were transfected with pBabe-puro-hp15 (a kind gift from Prof Gordon Peters, CRUK, London Research Institute) using Lipofectamine 2000 transfection reagent (Life Technologies, Carlsbad, CA) for 24 hours using 2 μl reagent and 1 μg of plasmid DNA per 500 μl as per the manufacturer’s guidance.

### FACS analysis

Between 0.5 and 1 × 10^6^ cells were grown in 6-well plates and treated as described. Cells were detached from the surface, pelleted, and washed twice in PBS before fixing with 70% ethanol for 1 hour at 4 °C, with the ethanol solution added while the cells were mixed on a vortex. Cells were stained with 1 μg/ml DAPI. A Canto II FACS (Beckton Dickinson, San Jose, CA) was used for sample analysis, according to manufacturer’s instructions, with 25,000 events captured per sample. Flow Jo software was used for analysis of the data output from the Canto II machine.

### Statistical analysis

All data are expressed as mean ± SEM except where specified. Student’s *t*-test was applied to determine statistical significance or ANOVA (LSD test) where stated. In all cases, statistical significance was defined as *P* < 0.05. Minimally, three independent repeats of each experiment were performed.

### Data availability statement

The data supporting the results reported in this article were generated during the study and are available from the corresponding author upon reasonable request. The cell lines used are available from the Functional Genomics Cell Bank at St George’s (http://anatomy.sgul.ac.uk/pages/WTFGCB.htm).

## ORCIDs

Syamal Patel: http://orcid.org/0000-0001-9900-6979

Elena V. Sviderskaya: http://orcid.org/0000-0002-4177-8236

Christopher J. Wilkinson: http://orcid.org/0000-0002-7448-0938

## Conflict of Interest

The authors state no conflict of interest.

## Figures and Tables

**Supplementary Table S1:** Melanoma Cell Lines and Their Mutations

Cell line	p15^INK^	p16^INK^	BRAF	NRA	TP53	Other mutations/notes
Hermes I	+	–				p16-null melanocytes immortalized with hTERT
NOHM 1	+	+				Normal diploid human melanocyte strain
NOHM 4	+	+				Normal diploid human melanocyte strain
NOHM 5	+	+				Normal diploid human melanocyte strain
NOHM 6	+	+				Normal diploid human melanocyte strain
SGM2 (RGP)	+	–	V600E	WT		(BRAF V600E) Subtetraploid: 85, XXX, )1, )3, )6, )9, )9, der(13,14)(q10;q10
SGM3 (RGP)	+	+	V600E			(BRAF V600E) Tetraploid, translocations in 3p and 8q, unbalanced 3q and 4qd
SGM4 (RGP)	+	–	V600E			(BRAF V600E) 46, XX, add(7)(q?36), add(17)(q?25)
SGM5 (VGP)	–	–				NRAS Q61K 46, XY, der(9)t(9,13), +der(9)t(9,13), add(9)(p?24), )22
WM-35 (RGP)	–	–	V600E	WT	WT	Heterozygous for BRAF
WM 1650 (RGP)	–	–				
WM-98-1 (VGP)	+	–	V600E	WT		BRAF V600E, not NRAS Q61K, this study
ME 10538 (VGP)	–	–	V600E			Homozygous for CDKN2A deletion
ME 1402 (VGP)	–	–	V600E			
WM-1341 B (VGP)	–	–				
DX3	–	–	WT	Q61R	WT	Activated N-RAS oncogene ATM p.Pro604Ser PTEN p.Lys128_Arg130del Parental, metastatic
A375P	–	–	V600E	WT	WT	Homozygous for CDKN2A p.Glu61Ter (c.181G>T) (p.Gly75Val; c.224G>T) and p.Glu69Ter Parental, metastatic

Abbreviations: RGP, radial growth phase; VGP, vertical growth phase; WT, wild-type.
